# Genetic Acquisition of NDM Gene Offers Sustainability among Clinical Isolates of *Pseudomonas aeruginosa* in Clinical Settings

**DOI:** 10.1371/journal.pone.0116611

**Published:** 2015-01-30

**Authors:** Shweta Mishra, Supriya Upadhyay, Malay Ranjan Sen, Anand Prakash Maurya, Debarati Choudhury, Amitabha Bhattacharjee

**Affiliations:** 1 Department of Microbiology, Institute of Medical Sciences, Banaras Hindu University, Varanasi, 221005, India; 2 Department of Biotechnology and Bioinformatics, North Eastern Hill University, Shillong, Meghalaya, India; 3 Department of Microbiology, Assam University, Silchar, 788011, India; 4 Department of Life Science and Bioinformatics, Assam University, Silchar, 788011, India; University of Malaya, MALAYSIA

## Abstract

New Delhi metallo β-lactamases are one of the most significant emerging resistance determinants towards carbapenem drugs. Their persistence and adaptability often depends on their genetic environment and linkage. This study reports a unique and novel arrangement of *bla*_NDM-1_ gene within clinical isolates of *Pseudomonas aeruginosa* from a tertiary referral hospital in north India. Three NDM positive clonally unrelated clinical isolates of *P. aeruginosa* were recovered from hospital patients. Association of integron with *bla*_NDM-1_ and presence of gene cassettes were assessed by PCR. Genetic linkage of NDM gene with IS*Aba125* was determined and in negative cases linkage in upstream region was mapped by inverse PCR. In which only one isolate’s NDM gene was linked with IS*Aba125* for mobility, while other two reveals new genetic arrangement and found to be inserted within DNA directed RNA polymerase gene of the host genome detected by inverse PCR followed by sequencing analysis. In continuation significance of this novel linkage was further analyzed wherein promoter site detected by Softberry BPROM software and activity were assessed by cloning succeeding semi-quantitative RT-PCR indicating the higher expression level of NDM gene. This study concluded out that the unique genetic makeup of NDM gene with DNA-dependent-RNA-polymerase favours adaptability to the host in hospital environment against huge antibiotic pressure.

## Introduction

The New Delhi Metallo-β-lactamase (NDM) has emerged as a major carbapenemase with rapid dissemination worldwide [1]. Clinical isolates harbouring NDM gene are often referred as superbugs. The NDM gene has been identified usually in enterobacteriaceae and recently in *Pseudomonas* spp. [2] which expresses resistance towards carbapenems and represents a significant threat for clinicians. This situation has been further complicated by the association of NDM gene with other resistant determinants [3]. Most importantly, however they represent an important stage in the evolution of antibiotic era.

Transfer of the *bla*_NDM_ among promiscuous plasmids is the major dissemination route including clonal outbreaks. Noticeably, *bla*_NDM_ has been frequently identified from unrelated gram negative bacilli, harboured by different plasmid types [4]. However, the mechanism of multiresistance trait of NDM positive isolates remained wanted and speculated that it could have been captured from original chromosomal location by mobile genetic elements. It has been reported that the *bla*_NDM_ can be carried by different plasmid types (IncA/C, IncF, IncL/M, or untypable) and occasionally found to be chromosomally integrated [5]. The sequence of few plasmids carrying *bla*_NDM_ are now available and reveals its inimitable genetic features such as association with insertion sequences and transposons for high mobility, acquisition of other multiresistance regions (*aadB, dfrA12, bla*_OXA-30_, *aacA4*) including additional important enzymes which make it more versatile [6].

In this study we have presented a unique genetic makeup of *bla*_NDM_ gene among clinical isolates along with other resistance factors. However, this novel finding suggests the insertion of *bla*_NDM_ within DNA directed RNA polymerase gene, thus favouring its survivability within hospital.

## Materials and Methods

### Bacterial isolates & Carbapenem susceptibility

A total of 105 consecutive, non-duplicates, carbapenem non-susceptibile isolates of *P.aeruginosa* were collected from indoor patients of Sir SunderLal Hospital, Banaras Hindu University, Varanasi, India, during March 2011 to September 2011. Identification of organisms was done by the conventional methods [7].

The metallo β-lactamase status of the strains was established by the Imipenem—Ethylene diamine tetra-acetic acid (EDTA) disc potentiation method [8]. A previously confirmed and sequenced clinical isolate of *E. coli* harbouring *bla*_NDM_ was taken as positive control [9] and *E. coli* ATCC 25922 was used as negative control.

All MBL positive isolates were suspended in 1mL of buffered peptone water supplemented with 30% glycerol (peptone glycerol) and were kept at −80°C. The isolates were also stored in minimal media (10% Peptone, 5% Sodium Chloride and 0.8% Agar) as stab culture. ERIC-PCR was performed for genotyping by using their respective primers to determine clonal relatedness of the isolates [10].

### Ethics Statement

This work and obtained samples specifically for this study has been ethically approved by the chairperson of the ethical committee of Institute of Medical Sciences, Banaras Hindu University, Varanasi; Ref. No.-Dean/2012-13/114.

### Genotypic detection of bla_NDM_ and their association with mobile genetic elements

Genomic DNA for PCR was extracted by using QIAamp DNA mini kit (Qiagen, Germany). Genotypic detection of *bla*_NDM_ was performed by PCR assay in all the MBL positive isolates. Reaction conditions and primers used for PCR amplification were as described earlier [11]. Consecutively, the presence of integrons were examined by PCR, using primers to amplify a 160 bp fragment for class 1 *integrase*, and 288 bp fragment for class 2 *integrase*. The primers, PCR conditions and reaction mixtures used were as described previously [12]. To find the genetic association of *bla*_NDM_ gene with integrons, PCR was performed using forward primer of the conserved region (5’CS) of integron gene and reverse primer of the characterized *bla*_NDM_ gene [12]. Integrons contain multiresistant regions known as gene cassettes which were determined by 59 base elements PCR (Table 1) [13].

**Table 1:** List of primers used in the study.

**Primers**	**Target**	**Sequence (5’–3’)**	**Reference**
HS287 F HS286 R	59be association	GGGATCCGCSGCTKANCTCVRRCGTTAGSC GGGATCCTCSGCTKGARCGAMTTGTTAGVC	12
Inv 1F Inv 1R	To amplify flanking region	ATGGAAACTGGCGACCAACGG AATCGTCGGGCGGATTTCACC	Present study
POL’F POL’R	To amplify flanking region	ACGTCAGAGCGATGAAGACG GACCTGGAACTGACCGTACG	Present study
NDM 3P	3’ end of linkage with DNA directed RNA Polymerase reverse	GATCGTGATGAGCCATTCCGCC	Present study
Native Pm Mutated Pm NDMc	To amplify RNA polymerase promoter along with integrated NDM gene	AACCTGATTGTCGAGCTCTACTC C**TC**GCTCTACTCCAAGTAAG**AA**C^*^ CATCGAAATCGCGCGATGGCAG	Present study

*underlined regions are mutated bases

Insertion sequences (IS), one of the major genetic elements in transposition of antibiotic resistance genes. In order to assess the linkage of NDM gene with the insertion sequence in our study, PCR analysis was performed by using forward primer of IS*Aba*125 (*Acinetobacter* specific), and reverse primer of NDM gene. The reaction mixture and running conditions were as described earlier [14].

### Detection of Novel linkage by inverse PCR

In order to determine the genetic environment surrounding the *bla*_NDM-1_ gene (apart from their linkage with IS*Aba*125 and integrons) inverse PCR was performed. DNA was extracted from the three strains and digested with Sau3AI (Biolabs, US). DNA fragments obtained were then autoligated at 16°C with T4 DNA ligase in higher dilution to allow self circularization. The fragment of DNA containing the *bla*_NDM-1_ gene was used as a template for an inverse PCR with primers designed from the *bla*_NDM-1_ gene sequence. Inv1F as forward and Inv1R as reverse primer [Table 1] were used for amplification. Reaction mixture was approximately 5 ng of template DNA, 10 pmol of each primer, 200 μM deoxynucleoside triphosphate (dNTP) mix, 2 mM MgCl2 and of Taq DNA polymerase in the reaction buffer supplied with the enzyme. Reaction conditions were: initial denaturation at 94°C for 3 min followed by 32 cycles at 94°C for 30 s, 58°C for 45 s, 72°C for 1 min 25 s, and final extension at 72°C for 8 min.

Once the sequence of amplified product of inverse PCR was obtained, the linkage was confirmed by using POL’F 5′ACGTCAGAGCGATGAAGACG-3′ as forward primer and Inv1R 5′AATCGTCGGGCGGATTTCACC-3′ as reverse primer to detect 5’ region of target site, followed by second PCR reaction with NDM 3PF 5′GATCGTGATGAGCCATTCCGCC3′ as forward primer and POL’R 5′GACCTGGAACTGACCGTACG3′ as a reverse primer to confirm 3’ target region. To, further establish this linkage, the PCR reaction was performed using POL’F as forward and POL’R as reverse primer among NDM positive isolates.

### Southern Hybridization

Simultaneously, to validate our study Southern blotting was performed on agarose gel by in-gel hybridization [15] with the *bla*_NDM_ probe labelled with DIG HIGH PRIME LABELING MIX (ROCHE, Germany) detection Kit. The digoxigenin-labeled *bla*_NDM_ specific probe was prepared using primers (Forward NDM 5′GGGCAGTCGCTTCCAACGGT 3′ and Reverse 5′CGACCGGCAGGTTGATCTCC 3′) that amplify a 130 bp region of the *bla*_NDM_ gene. Total genomic DNA was digested by HindIII for fragmentation of DNA which is followed by the transfer to nylon membrane (Hybond N, Amersham, UK) and then hybridised with prepared *bla*_NDM_ specific probe. Detection was performed by using an NBT color detection kit. (ROCHE,Germany). Promoter activity was assessed by designing two sets of primers; in first set forward primer (RNAPROM) was designed from upstream region of the RNA polymerase promoter whereas in the second set the primer with desired mutation was generated (MUTPROM) in the promoter region. For both the sets the reverse primer used was NDMc [Table 1]. The PCR amplicons were sequenced to confirm and cloned using p^GEM-T^ vector (Promega, Germany) in *E. coli* JM107. Further, RNA was isolated from both the constructs using RNeasy^R^ Mini Kit (Qiagen Hilden, Germany) and cDNA was prepared using Quanti Tect Reverse Transcription kit (Qiagen, Hilden, Germany). Thereafter, Semi quantitative reverse transcriptase PCR was performed to determine the expression of *bla*_NDM_ in both the constructs.

### Transferability & plasmid profiling

Horizontal transferability of *bla*_NDM_ was investigated by transformation assay. Conjugation experiment was carried out between clinical isolates as donors and streptomycin resistance *E.coli* recipient strain B (Genei, Banglore, India). Overnight culture of bacteria were diluted in Luria Bertani broth (Hi-Media, Mumbai, India) and was grown at 37°C till the O.D. of the recipient and donor culture reached 0.8–0.9 at A_600_. Donor and recipient cells were mixed at 1:5 donor-to-recipient ratios and transconjugants were selected on imipenem (0.25mg/L) + streptomycin (1000mg/L) agar plates. Transformation was carried out using *E.coli* JM107 as recipient. Transformants were further selected on imipenem (0.25mg/L) containing LB agar plates.

For the detection of incompatibility group type of plasmid in transformants with *bla*_NDM_ as well as in *bla*_NDM_ harbouring donor strains, PCR based replicon typing was carried out targeting 18 different replicon types [16]. Plasmid stability and fitness was also assessed by serial passage of *bla*_NDM_ positive isolates on LB broth without any antibiotic pressure. After each passage the isolates were tested for the presence of *bla*_NDM_ PCR assay.

### Detection of other resistant determinants

To investigate the presence of other resistant determinants along with NDM, isolates were further tested for the co-existence of other MBL genes such as *bla*_IMP_ and *bla*_VIM_, as well as ESBLs, AmpC and class D carbapenemase (OXA-48, OXA-58, OXA-23 and OXA-198) genes by multiplex PCR [17–23]. In addition, the study of efflux pump activity of the strains was phenotypically detected by double disc synergy test using meropenem and CCCP (carbonyl cyanide m-chlorophenylhydrazone) [24]. Consequently the expression of the Mex-efflux system was determined by quantitative real time PCR assay. PA01 strain was used as control [25].

### Antibiotic susceptibility testing

Antimicrobial sensitivity testing was performed on Mueller-Hinton agar (Hi-Media, Mumbai, India) plates by Kirby Bauer disc diffusion method and interpreted as per CLSI recommendations [26]. The antibiotic tested were amikacin (30μg), gentamicin (10μg), netilmicin (30μg), tobramicin (10μg), ceftazidime (30μg), ciprofloxacin (5μg), imipenem (10μg), meropenem (10μg), piperacillin/tazobactum (100/10μg) and polymyxin B (300μg) (Hi-Media, Mumbai, India). MICs of all the isolates were determined by the agar dilution method against cefotaxime, ceftazidime, ceftriaxone (Hi-Media, Mumbai, India), cefepime (Alembic Ltd., Vadodara, India), aztreonam (Aristo Pharmaceuticals Ltd., Mumbai, India), imipenem (United Biotech, Solan, India), meropenem (AstraZeneca Pharmaceuticals Ltd., Bangalore, India), tigecycline (Taj Pharmaceuticals Ltd., Mumbai, India), polymyxin (Celon laboratories Ltd, Andhra Pradesh, India). *Escherichia coli* ATCC 25922 was used as a control.

### Sequencing analysis

All the PCR amplicons were purified using the QIAquick Gel Extraction kit (QIAGEN Inc., Valencia, CA) and were subjected to DNA sequencing (Merck, Bangalore, India). Sequences were analyzed using the BLAST suite of programs. (http://www.ncbi.nlm.nih.gov/BLAST/). Further, promoter sites were also determined by using Soft Berry BPROM software (http://linux1.softberry.com/berry.phtml?topic=bprom&group=programs&subgroup=gfindb).

## Results

### Genetic context of *bla*_NDM_

Among the 105 isolates, 38 strains exhibited MBL activity by Imipenem EDTA disc potentiation method, of which three nonclonal isolates (PA6, PA38 and PA47) were found to harbour *bla*_NDM-1_ (Table 2) and were carrying class 1 integron too. While out of the 35 MBL positive but *bla*_NDM_ negative isolates, 7 and 3 isolates were harbouring VIM and IMP gene respectively. Remaining phenotypically MBL positive isolates did not showed any amplification with the targeted MBL gene primers (IMP, VIM, NDM). Further, class 1 integron was detected among the 99/105 (94.28%) isolates. None of the study isolates were found carrying class 2 integrase. On performing the ERIC PCR for all the 105 isolates a total of 46 different clonal types were observed. However two non MBL strains were found to be clonal with PA47.

**Table 2:** Clinical details of the isolates harbouring NDM gene.

**ID no.**	**Isolate**	**Patient age/Sex**	**Clinical specimen**	**Ward/OPD/ICU**	**Current diagnosis**	**Carbapenem Susceptibility**	**Other susceptible antibiotics tested *in vitro***	**Coproduction of Class C**	**NDM or other MBL genes**
PA6	*P.aeruginosa*	24year/female	Tissue	Burn	sepsis	R	Polymyxin B	CMY-2	NDM
PA38	*P.aeruginosa*	25 year/female	Central tip	FICU	VAP	R	Polymyxin B	ACT, CMY-2	NDM
PA47	*P.aeruginosa*	29 year/female	Pus	Ortho	sepsis	R	Polymyxin B	DHA, ACT	NDM,IMP, VIM

OPD, outpatient department; ICU, Intensive care unit; VAP, ventilator-associated pneumonia; FICU, Female intensive care unit R, resistant; Class C-Plasmid mediated AmpC β -lactamases.

**Figure 1:**
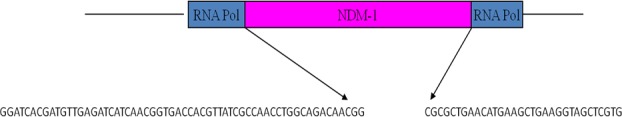
Schematic representation of genetic arrangement of *bla*_NDM_ in *Pseudomonas aeruginosa (PA38 & PA47)*. Section of DNA directed RNA polymerase gene in blue colored block while inserted NDM gene in pink block. NDM adjacent segments of DNA sequence were shown below.

**Figure 2:**
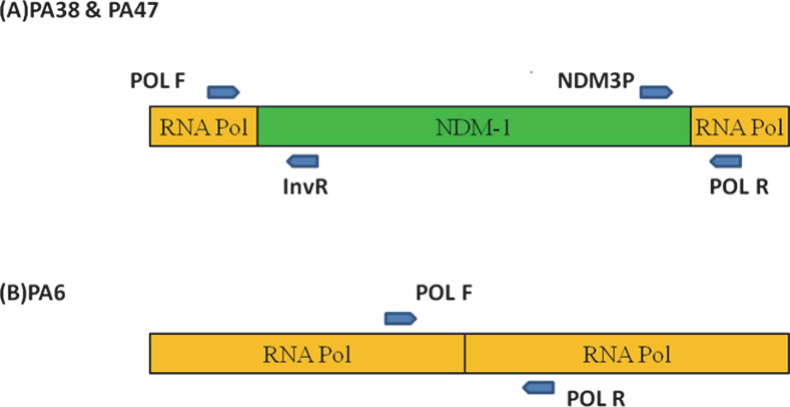
Comparative analysis of PCR amplified product in different *P. aeruginosa* strains. (A) NDM gene inserted in DNA directed RNA polymerase. (B) There is no insertion of NDM gene in RNA polymerase gene as intact gene segment of DNA directed RNA polymerase were detected through PCR mapping.

**Figure 3:**
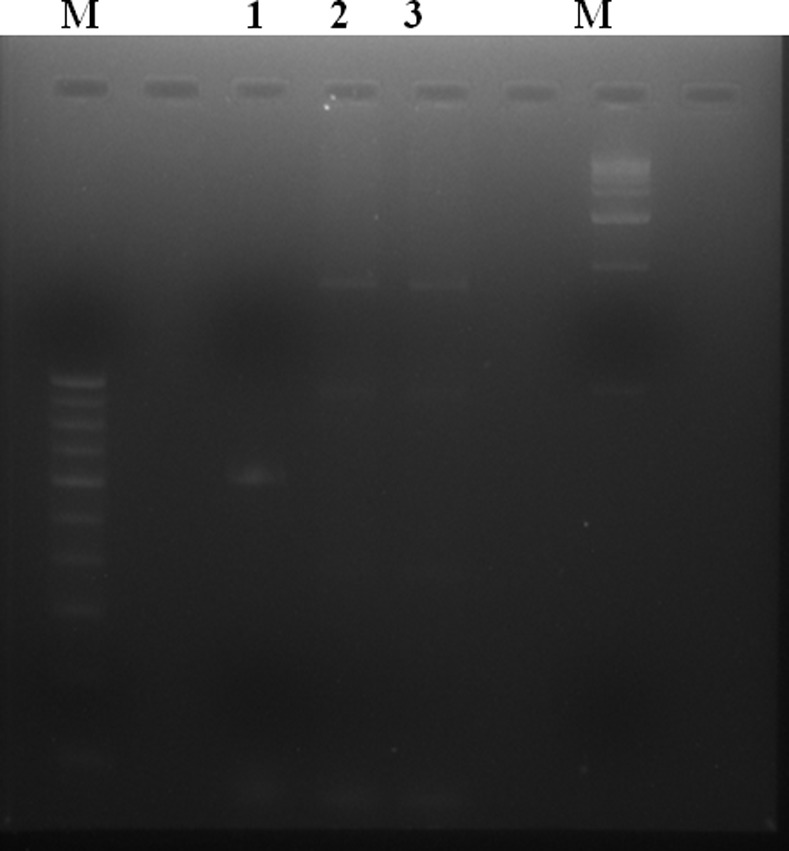
DNA directed RNA polymerase gene amplification in three NDM harbouring isolates. Lane 1- PA6 showing expected amplification of the intact gene; Lane 2 & 3- PA38 & 47 showing amplification of the gene along with the NDM insert (amplified product was comparatively of higher basepair than PA6).

**Figure 4:**
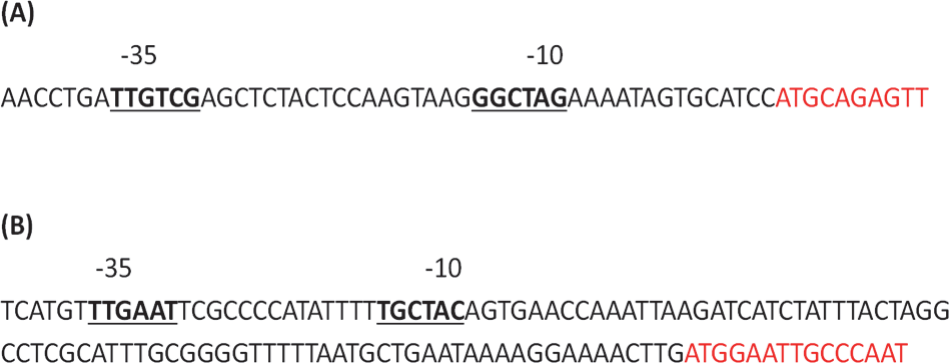
Promoter sequence for NDM gene as determined by Softberry BPROM software. (A) Promoter sequence of *bla*NDM within RNA polymerase gene in *P. aeruginosa* strain (AF047025) and (B) NDM promoter in *A. baumannii* associated with some insertion sequence (HQ857107). The −35 and −10 motifs of promoter are in boldface type whereas ORF sequence in red color font.

**Figure 5:**
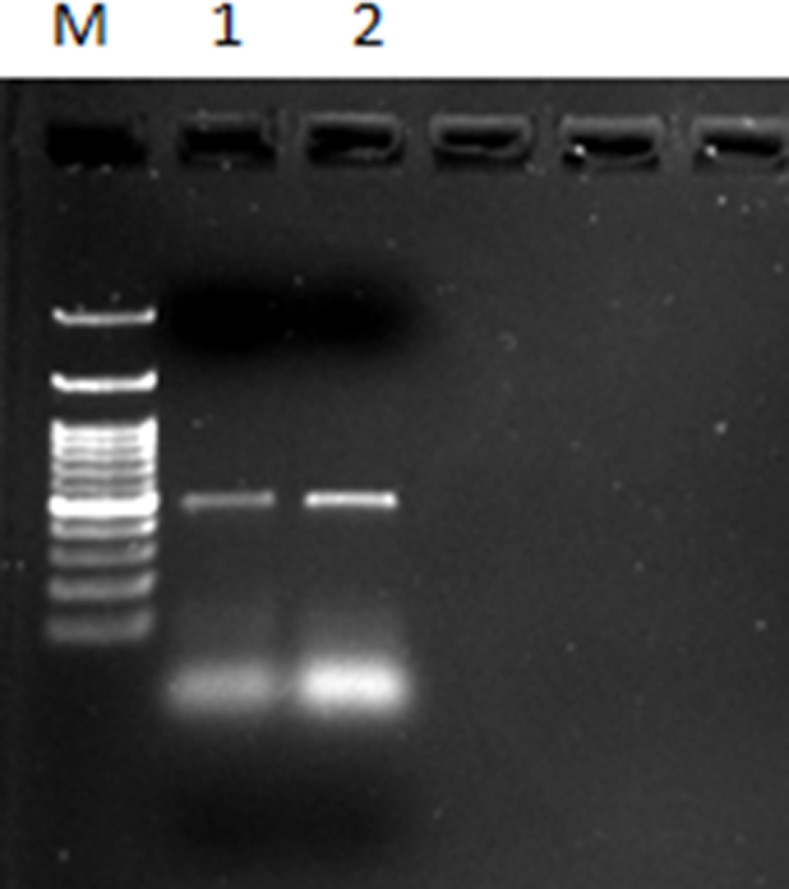
Semi quantitative reverse transcriptase PCR products showing promoter activity for expression of NDM gene, Lane1-NDM gene with mutated promoter (Low expression); Lane2- NDM with intact promoter showing higher expression.

However, among the three NDM harbouring isolates 59be PCR amplification and their sequencing revealed presence of dihydrofolate reductase (*dhfr*) and aminoglycoside acetyl transferase (*aac(6’)*) within the gene cassette. While analysing the upstream linkage of NDM gene with IS*Aba125*, isolate PA6 formed a band of 850 bp thus showing the association between them which was confirmed by sequencing. However, in case of PA38 and PA47 inverse PCR result gave a novel finding that *bla*_NDM_ gene got integrated within DNA dependent RNA polymerase of host genome. This was further confirmed by designing primers which could amplify both 5’ and 3’ region of the insert (Fig. 1). This insertion was further verified and supported by the fact that the amplified product of higher basepair with Pol’F and Pol’R could be observed in PA38 and PA47 while expected size of amplification was evident with PA6 (Figs. 2 and 3). It was also observed that the recombinant *bla*_NDM_ used promoter region of DNA dependent RNA polymerase for its expression (Fig. 4). Further, Semi quantitative reverse transcriptase PCR also establishes DNA dependent RNA polymerase promoter activity for higher expression of NDM gene (Fig. 5).

Results of Southern hybridization were evident to suggest genetic background of the *bla*_NDM_-_1_. Plasmids of all three isolates were transferred to the nylon membrane while only PA6 hybridized with NDM-1 specific probe. Whereas when the same experiment was done with total DNA of all the test isolates, it was observed that all of them hybridized with the probe showing identical banding pattern with PA38 and PA47. Transformation and conjugation was successful only with PA6, where transconjugant showed to carry *bla*_NDM_ within the 30–50kb plasmid. However, all the three isolates were carrying the plasmid of *Inc*T type. The plasmid was highly unstable and was lost after six consecutive passages without any antibiotic in LB broth.

### Coproduction of other resistant determinants

All the three isolates were negative for ESBLs but coproducing AmpC β-lactamase, while PA 47 was carrying *bla*_IMP_ and *bla*_VIM_ along with NDM gene. All of them were harbouring class D carbapenemase as well (Table 2). On performing the quantitative real time PCR to assess the level of transcription in MexAB-OprM efflux pump system, it was observed that the transcription level was 16-fold higher while under inducing condition when compared with PA01.

### Antimicrobial susceptibility

On observing the antimicrobial susceptibility of these NDM producers, the isolates showed resistance towards all the tested antibiotics namely; cephalosporins, aztreonam, carbapenems, aminoglycoside and fluoroquinolones but were susceptible against polymyxin B. MIC of carbapenem was much higher for PA38 and PA47 compare to PA6 (Table 3).

**Table 3:** MIC (mg/L) of *P. aeruginosa* against cephalosporins and carbapenems.

**Isolate ID**	**CTX**	**CAZ**	**CEP**	**CFT**	**ERTA**	**IMP**	**MER**
PA6	≥512	≥512	≥512	≥512	256	128	64
PA38	≥512	≥512	≥512	≥512	512	512	512
PA47	≥512	≥512	≥512	≥512	512	512	512

MIC, Minimum Inhibitory Concentration; CTX, Cefotaxime; CAZ, Ceftazidime; CEP, Cefepime; CFT Ceftriaxone; ERTA, Ertapenem; IMP,Imipenem; MER, Meropenem.

## Discussion

Among the carbapenemases, the NDM gene has gained particular attention due to its global dissemination and multidrug resistance phenotype [27]. In the study, we found that all the three *bla*_NDM_ harbouring strains were highly resistant to the antibiotics tested except polymyxin. Although the MBL enzymes donot affect monobactam, the co-existence of different AmpC genes such as *bla*_EBC,_ *bla*_DHA_ and *bla*_CIT_ in these isolates were found to confer resistance to monobactam [14, 28]. Besides harbouring *bla*_NDM_ the isolates were also carrying class D carbapenemases along with intrinsic mechanism. Similar genotype was earlier reported from India [29]. However, it leaves scope for future studies to address the exact role of six different carbapenem resistant determinants in a single isolate (PA47) when exposed to carbapenems.The study could demonstrate that *bla*_NDM-1_ in PA6 is plasmid mediated which was transferable by conjugation. However, same incompatibility type plasmid (lacking *bla*_NDM-1_) was present in other two isolates (PA38 and PA47). So, this plasmid could play the role of carrier for their acquisition of resistant determinant which later got integrated within the host genome.

In the previous study it was reported that the NDM gene has originated from *Acinetobacter baumannii* and is linked with IS*Aba*125 in the upstream region. However, during horizontal transfer the gene got excised from the *Acinetobacter* DNA along with whole or truncated portion of IS*Aba125* [30]. Therefore *Acinetobacter* specific primer was used for the study and it was observed that only PA6 showed the presence of this insertion sequence in the upstream area. But acquisition of *bla*_NDM_ within the DNA dependent RNA polymerase of host chromosome is quite unique and not reported previously.

The new genetic makeup of *bla*_NDM_ under the control of RNA polymerase promoter might be responsible for higher level of expression, which could probably be the explanation for high MIC of PA38 and PA47.

The clinical challenge posed by *bla*_NDM_ is currently higher worldwide due to its potential transferability. As our findings also suggest that the unique genetic makeup of *bla*_NDM_ is for make an ease to endure the isolate within hospital environment to counter increasing antibiotic pressure. This is an important consideration that *Pseudomonas* with recombinant *bla*_NDM_ is crucial for the hospital infection management and therapeutic options, otherwise this new genetic adaptability will lead in to more serious public health implications. From the study it cannot be truly predicted whether this recombination was a by chance event or a true event since the sample size was too small. Therefore, further investigation is advocated to establish this fact.

## References

[1] Nordmann P, Poirel L, Toleman MA, Walsh TR, Livermore DM (2011) The emerging carbapenemases. Trends microbial 19: 588–595. 10.1016/j.tim.2011.09.005 22078325

[2] Janvier F, Jeannot K, Tessé S, Nicoud MR, Delacour H, et al. (2013) Molecular characterization of *bla*NDM-1 in a ST235 *Pseudomonas aeruginosa* isolate, France. Antimicrob Agents Chemother 57: 3408–3411. 23612200 10.1128/AAC.02334-12 PMC3697391

[3] Dortet L, Nordmann P, Poirel L (2012) Association of the emerging carbapenemase NDM-1 with a bleomycin resistance protein in enterobacteriaceae and *Acinetobacter baumannii* , Antimicrob Agents Chemother 56: 1693–1697. 10.1128/AAC.05583-11 22290943 PMC3318356

[4] Poirel L, Dortet L, Bernabeu S, Nordmann P (2011) Genetic feature of *bla*NDM-1 positive enterobacteriaceae. Antimicrob Agents Chemother 55: 5403–5407. 21859933 10.1128/AAC.00585-11 PMC3195013

[5] Nordmann P, Dortet L, Poirel L (2012) Carbapenem resistance in Enterobacteriaceae: here is the storm. Trends Mol Med 18: 263–272. 10.1016/j.molmed.2012.03.003 22480775

[6] Patridge SR, Iredell JR (2012) Genetic context of *bla*NDM-1. Antimicrob Agents Chemother 56: 6605–6607.

[7] Collee JG, Miles RS, Watt B (1996) Tests for identification of bacteria. In Mackie & McCartney Practical Medical Microbiology (Edited by Collee JG, Marmion BP, Fraser AG, Simmons A). Churchill Livingstone, New York 131–149.

[8] Yong D, Lee K, Yum JH, Shin HB, Rossolini GM, et al. (2002) Imipenem–EDTA disk method for differentiation of metallo-β-lactamase-producing clinical isolates of *Pseudomonas* spp. and *Acinetobacter* spp. J Clin Microbiol 40: 3798–3801. 10.1128/JCM.40.10.3798-3801.2002 12354884 PMC130862

[9] Kumari S, Sen MR, Upadhyay S, Bhattacharjee A (2011) Dissemination of the New Delhi Metallo-β-lactamase-1 (NDM-1) among *Enterobacteriaceae* in a tertiary referral hospital in north India. J Antimicrob Chemother: 66(7):1646–47. 10.1093/jac/dkr180 21596721

[10] Versalovic J, Koueth T, Lupski JR (1991) Distribution of repetitive DNA sequences in eubacteria and application to fingerprinting of bacterial genomes. Nucl Acid Res 19: 6823–6831. 10.1093/nar/19.24.6823 1762913 PMC329316

[11] Yong D, Toleman MA, Giske CJ, Cho HS, Sundman K, et al. (2009) Characterization of a new metallo-β-lactamase gene, *bla*NDM-1, and a novel erythromycin esterase gene carried on a unique genetic structure in *Klebsiella pneumoniae* sequence type 14 from India. Antimicrob Agents Chemother 53: 5046–5054. 10.1128/AAC.00774-09 19770275 PMC2786356

[12] Koeleman JGM, Stoof J, Vanderbij MW, Grauls V, Savelkoul P (2001) Identification of epidemic strains of *Acinetobacter baumannii* by *Integrase* Gene PCR. J Clin Microbiol 39: 8–13. 10.1128/JCM.39.1.8-13.2001 11136740 PMC87671

[13] Stokes HW, Holmes AJ, Nield BS, Nield BS, Holley MP, et al. (2001) Gene cassette PCR: Sequence-independent recovery of entire genes from environmental DNA. Appl Environ Microbiol 67:5240–5246. 10.1128/AEM.67.11.5240-5246.2001 11679351 PMC93296

[14] Mishra S, Sen MR, Upadhyay S, Bhattacharjee A (2013) Genetic linkage of *bla*NDM among nosocomial isolates of *Acinetobacter baumannii* from a tertiary referral hospital in north India. Int J Antimicrob Agents 41: 452–456. 10.1016/j.ijantimicag.2013.01.007 23434534

[15] Sambrook J, Fritsch EF, Maniatis T (1989) Molecular cloning: a laboratory manual. 2nd ed. Cold Spring Harbour, N.Y: Cold Spring Harbour Laboratory Press p32.

[16] Carattoli A, Bertinia A, Villaa L, Falbo V, Hopkins KL, et al. (2005) Identification of plasmids by PCR-based replicon typing. J Microbiol Methods 63: 219–228. 10.1016/j.mimet.2005.03.018 15935499

[17] Senda K, Arakawa Y, Ichiyama S, Nakashima K, Ito H, et al. (1996) PCR detection of metallo-β-lactamase gene (*bla*IMP) in gram-negative rods resistant to broad spectrum β- lactams. J. Clin Microbiol 34: 2909–2913. 8940421 10.1128/jcm.34.12.2909-2913.1996 PMC229432

[18] Tsakris A, Pournaras S, Woodford N, Palepou MF, Babini GS, et al. (2000) Outbreak of infections caused by *Pseudomonas aeruginosa* producing VIM-1 carbapenemase in Greece. J Clin Microbiol 38: 1290–1292. 10699045 10.1128/jcm.38.3.1290-1292.2000 PMC88610

[19] Javier PF, Hanson ND (2002) Detection of plasmid-mediated AmpC β-lactamase genes in clinical isolates by using multiplex pcr. J Clin Microbiol 40:2153–2162. 10.1128/JCM.40.6.2153-2162.2002 12037080 PMC130804

[20] Bhattacharjee A, Sen MR, Prakash P, Anupurba S (2008) Role of beta-lactamase inhibitors in enterobacterial isolates producing extended-spectrum beta-lactamases.J Antimicrobl Chemother 61: 309–314. 10.1093/jac/dkm494 18174199

[21] Jeong SH, Bae IK, Park KO, An YJ, Sohn SG, et al. (2006) Outbreaks of imipenem335 resistant *Acinetobacter baumannii* producing carbapenemases in Korea. J Microbiol 44: 423–431. 16953178

[22] Dallenne C, Costa AD, Decre D, Favier C, Arlet G (2010) Development of a set of multiplex PCR assays for the detection of genes encoding important β-lactamases in enterobacteriaceae. J Antimicrob Chemother 65: 490–495. 10.1093/jac/dkp498 20071363

[23] Poirel L, Marque S, He´ritier C, Segonds C, Chabanon G, et al. (2005) OXA-58, a novel class D β-lactamase involved in resistance to carbapenems in *Acinetobacter baumannii* . Antimicrob Agents Chemother 49: 202–208. 10.1128/AAC.49.1.202-208.2005 15616297 PMC538857

[24] Quale J, Bratu S, Landman D, Heddurshetti R (2003) Molecular Epidemiology and Mechanisms of Carbapenem Resistance in *Acinetobacter baumannii* Endemic in New York City. Clin Infect Dis 37: 214–220. 10.1086/375821 12856214

[25] Mesaros N, Glupczynski Y, Avrain L, Caceres NE, Tulkens PM, et al. (2007) A combined phenotypic and genotypic method for the detection of Mex efflux pumps in *Pseudomonas aeruginosa* . J Antimicrob Chemother 59: 378–386. 10.1093/jac/dkl504 17289770

[26] CLSI (2005) Performance standards for antimicrobial disc susceptibility test. CLSI: Wayne PA – M100-S15

[27] Kumaraswamy KK, Toleman MA, Walsh TR, Bagaria J, Butt F, et al. (2010) Emergence of a new antibiotic resistance mechanism in India, Pakistan, and the UK: a molecular, biological and epidemiological study. Lancet Infect Dis 10: 597–602. 10.1016/S1473-3099(10)70143-2 20705517 PMC2933358

[28] Berçot B, Poirel L, Dortet L, Nordmann P (2011) *In vitro* evaluvation of antibiotic synergy for NDM-1 producing enterobacteriaceae. J Antimicrob Chemother 66: 2295–2297. 10.1093/jac/dkr296 21807739

[29] Kumarasamy K, Thirunarayan MA, Krishnan P (2010) Coexistence of *bla*OXA-23 with *bla*NDM-1 and armA in clinical isolates of *Acinetobacter baumannii* from India. J Antimicrob Chemother 65: 2253–2254. 10.1093/jac/dkq273 20650909

[30] Toleman MA, Spencer J, Jones L, Walsh TR (2012) *bla*NDM-1 is a chimera likely constructed in *Acinetobacter baumannii* . Antimicrob Agents Chemother 56: 2773–2776. 10.1128/AAC.06297-11 22314529 PMC3346620

